# Malaria attributable fractions with changing transmission intensity: Bayesian latent class vs logistic models

**DOI:** 10.1186/s12936-022-04346-9

**Published:** 2022-11-11

**Authors:** Kennedy Mwai, Irene Nkumama, Amos Thairu, James Mburu, Dennis Odera, Rinter Kimathi, Lydia Nyamako, James Tuju, Samson Kinyanjui, Eustasius Musenge, Faith Osier

**Affiliations:** 1 Epidemiology and Biostatistics Division, School of Public Health, University of the Witwatersrand, Johannesburg, South Africa; 2 Centre for Geographic Medicine Research (Coast), Kenya Medical Research Institute-Wellcome Trust Research Programme, Kilifi, Kenya; 3 Centre of Infectious Diseases, Parasitology, Heidelberg University Hospital, Heidelberg, Germany; 4 Department of Biotechnology and Biochemistry, Pwani University, Kilifi, Kenya; 5 Centre for Tropical Medicine and Global Health, Nuffield Department of Clinical Medicine, University of Oxford, Oxford, UK

**Keywords:** Case definition, Malaria, Attributable fractions, Probability

## Abstract

**Background:**

Asymptomatic carriage of malaria parasites is common in high transmission intensity areas and confounds clinical case definitions for research studies. This is important for investigations that aim to identify immune correlates of protection from clinical malaria. The proportion of fevers attributable to malaria parasites is widely used to define different thresholds of parasite density associated with febrile episodes. The varying intensity of malaria transmission was investigated to check whether it had a significant impact on the parasite density thresholds. The same dataset was used to explore an alternative statistical approach, using the probability of developing fevers as a choice over threshold cut-offs. The former has been reported to increase predictive power.

**Methods:**

Data from children monitored longitudinally between 2005 and 2017 from Junju and Chonyi in Kilifi, Kenya were used. Performance comparison of Bayesian-latent class and logistic power models in estimating malaria attributable fractions and probabilities of having fever given a parasite density with changing malaria transmission intensity was done using Junju cohort. Zero-inflated beta regressions were used to assess the impact of using probabilities to evaluate anti-merozoite antibodies as correlates of protection, compared with multilevel binary regression using data from Chonyi and Junju.

**Results:**

Malaria transmission intensity declined from over 49% to 5% between 2006 and 2017, respectively. During this period, malaria attributable fraction varied between 27–59% using logistic regression compared to 10–36% with the Bayesian latent class approach. Both models estimated similar patterns of fevers attributable to malaria with changing transmission intensities. The Bayesian latent class model performed well in estimating the probabilities of having fever, while the latter was efficient in determining the parasite density threshold. However, compared to the logistic power model, the Bayesian algorithm yielded lower estimates for both attributable fractions and probabilities of fever. In modelling the association of merozoite antibodies and clinical malaria, both approaches resulted in comparable estimates, but the utilization of probabilities had a better statistical fit.

**Conclusions:**

Malaria attributable fractions, varied with an overall decline in the malaria transmission intensity in this setting but did not significantly impact the outcomes of analyses aimed at identifying immune correlates of protection. These data confirm the statistical advantage of using probabilities over binary data.

**Supplementary Information:**

The online version contains supplementary material available at 10.1186/s12936-022-04346-9.

## Background

Asymptomatic carriage of malaria parasites is highly prevalent in areas with high malaria transmission as a result of naturally acquired immunity [1]. It is, therefore, likely that, in such areas, an individual with a non-malarial fever has coincidental parasitaemia. Since the likelihood of having fever generally increases with parasite density, [1–3] the assumption is that fever in the presence of parasitaemia necessarily constitutes clinical malaria. However, in high transmission settings [4], parasitaemia accompanied by fever may not be adequate to define an episode of clinical malaria and may lead to differential misclassification. Besides causing an overestimation of malaria burden in an area [1, 5], the misclassification complicates immunological and clinical trials where clinical malaria cases are an endpoint or one of the outcome variables. As an outcome variable, it is particularly important for identifying correlates of protection from clinical episodes to inform vaccine development.

To overcome this problem of misdiagnosis, different studies have based the case definition of febrile malaria with parasite density above a locally defined threshold. The computation of malaria attributable fractions (MAF) or the proportion of fevers due to malaria parasites has been used to define different thresholds for parasitaemia [2, 3].

The classical method for deriving the attributable fraction is a simple numerator denominator approach [6] which is prone to bias when applied in high malaria transmission areas [5]. In high transmission settings, individuals may have parasites and not show clinical signs of malaria. Logistic regression models are typically used to handle this bias. The model determine the risk of the outcome as a continuous function of parasite density [1, 2] and have been widely used to obtain attributable fractions against a range of outcomes with parasitaemia as the exposure variable [1, 2, 7–9]. Additionally, a Bayesian latent class model of two-component mixture distributions was proposed to improve the estimation of attributable fractions [3]. The latent class model was developed to handle the limitation of imprecise or negative attributable fractions occasionally observed in standard logistic regression models [5].

Malaria transmission intensity has been found to strongly influence the attributable fractions. In a study conducted in two areas with different transmission intensities in Kilifi at the coast of Kenya, a MAF of 50.2% was estimated for Ngerenya, the low transmission site and 47.9% for Chonyi, the high transmission site. In the study, the logistic regression method was applied and derived a parasite density threshold of 2500 parasites/$\upmu$L of blood as the most appropriate to distinguish malaria-attributable fevers from fevers due to other causes in both settings. Following Ngerenya and Chonyi study, 2500 parasites/$\upmu$L threshold has been widely applied in the definition of malaria cases in various studies conducted along the Kenyan coast [7, 10–13].

Significant reductions in malaria transmission and admissions have been reported over the last decade in endemic countries in Africa [14] and in particular on the Kenyan coast [11, 15, 16]. Based on this observed reduction in transmission and the influence of transmission intensity on the MAF’s, the present study was conducted to determine the variation of malaria attributable fractions over time.

The probability of fever as a function of parasite density and the optimal parasite thresholds was estimated using logistic regression [3, 17]. The estimated probabilities of fever have been used in determining risk of developing clinical episodes in malaria vaccine trials. In these trials, the probabilities estimated from a Bayesian latent class model were proposed as a better approach to compare the placebo and control groups [18, 19].

Several articles [20–24] have pointed out problems associated with the categorization of data. These include not only the loss of information on variation and statistical power, but also an increased risk of type I errors and poor predictive performance [21, 22, 24]. This study also explores the utilization of probability estimates from Bayesian latent class models as an alternative to dichotomizing individuals using a selected parasite density threshold.

## Methods

### Study area and population

**Fig. 1:**
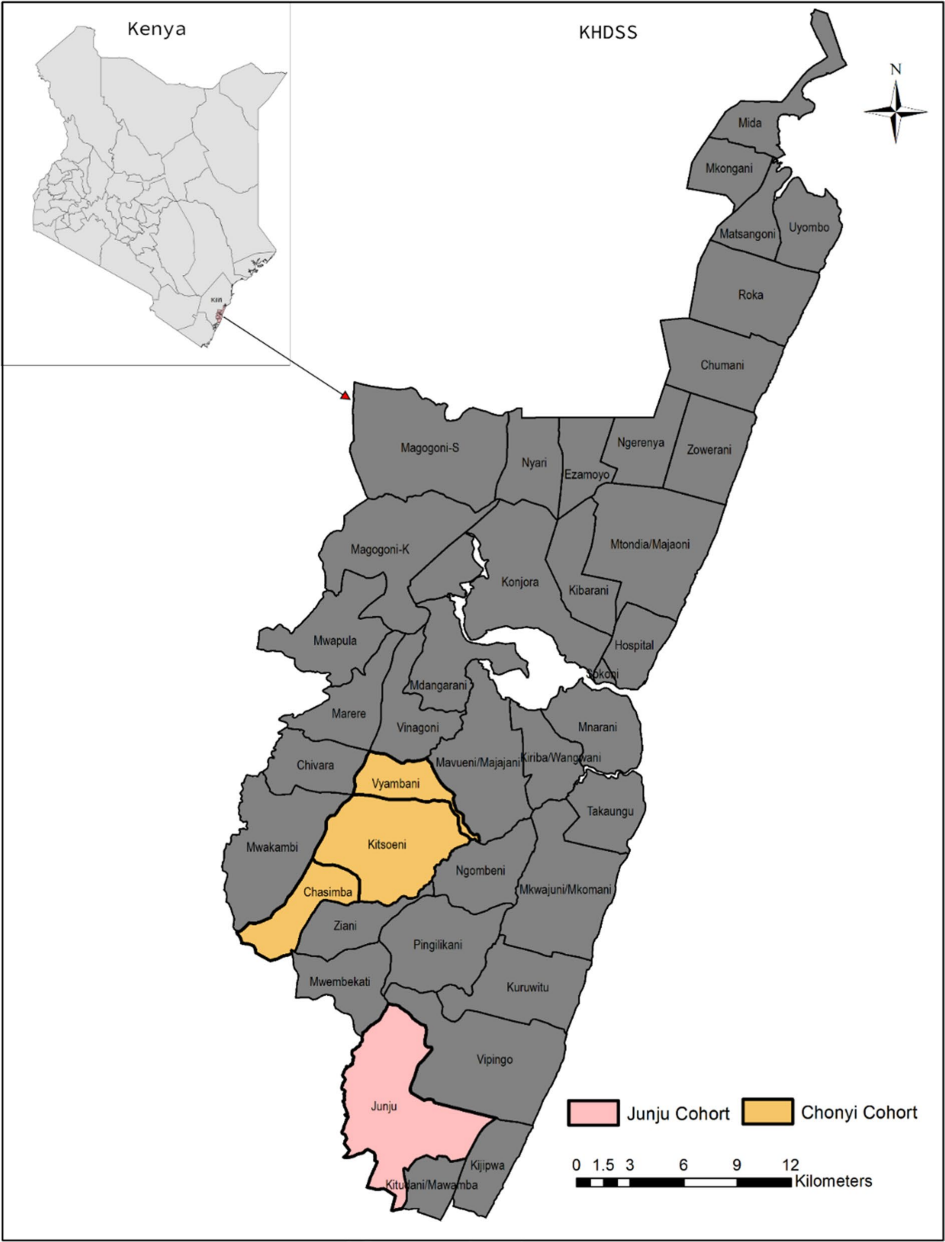
Junju and Chonyi study sites in the Kilifi Health and Demographic Surveillance System (KHDSS)

This research utilized cohort data from Junju and Chonyi sub-counties in Kilifi County, which is part of the Kilifi Health and Demographic Surveillance System (KHDSS) on the coastal region of Kenya Fig. 1 [33]. The area has two malaria transmission seasons May–July and November–December. For Junju the data were prospectively collected from participants aged 1 to 15 years old between 2005 and 2017 (inclusive) who were initially recruited into a malaria vaccine trial [34]. The Chonyi dataset had 286 children aged between 0–10 years collected in October 2000 and was only used for correlates of disease selection model comparison [26].

### Malaria parasite prevalence cross-sectional survey

A cross-sectional bleed survey was done every year at the beginning of the malaria season (March–May) for the Junju cohort as shown in Table 1 except for 2005 and 2006 when the surveys were done during the malaria season for a vaccine trial. For the Chonyi cohort, the cross-sectional malaria survey was conducted in October 2000. Parasitaemia was determined by thin smear microscopy. In both studies, the participants were followed up both actively with weekly home visits by trained field workers and passively at health facilities to identify clinical episodes of malaria. Blood smears were prepared to determine parasite densities for any child who had a fever (axillary temp ≥ 37.5 °C) for the cross-section surveys and follow-up surveillance, respectively. The Government of Kenya-recommended first-line treatment was used for treatment of malaria episodes.

**Table 1:** Prevalence of *Plasmodium falciparum* positivity, fever, and presumptive malaria (fever + parasitaemia) in the pre-transmission season cross-sectional survey and the number of active follow-up events in the Chonyi 2000 and Junju 2005–2007 Cohorts

Year	Samples (n)	Mean age in years (min–max)	P. *falciparum* (%)	Fever (%)	Presumptive malaria (%)	Active follow-up events (n)
2000^a^	286	5.62 (< 1–10)	42.31%	–	–	626
2005	372	3.9 (1–9.0)	49.05%	0.81%	0.54%	1029
2006	300	4.5 (1.5–9.5)	31.21%	0.67%	0.33%	615
2007	339	4.8 (< 1–11.0)	15.93%	3.24%	1.18%	1816
2008	341	5.4 (< 1–12.0)	29.62%	3.52%	3.52%	884
2009	352	5.8 (< 1–13.0)	20.17%	2.56%	1.14%	958
2010	377	6.5 (< 1–14.0)	27.59%	3.98%	2.65%	957
2011	377	7.1 (< 1–12.7)	23.08%	1.86%	1.33%	891
2012*	399	7.1 (< 1–13.7)	16.79%	1.75%	0.50%	696
2013	410	7.5 (< 1–14.7)	8.78%	1.46%	0.24%	1483
2014	404	8.2 (< 1–15.7)	14.25%	1.49%	0.50%	1619
2015	400	8.3 (< 1–16.5)	17.36%	1.25%	0.25%	1319
2016	316	7.8 (< 1–15.0)	11.61%	1.27%	-	1087
2017	335	7.1 (< 1–15.0)	4.32%	1.19%	-	1050

Participants were recruited for the original vaccine study in 2005 and 2006 and sampling of participants extended into the high transmission season each year

^*^Excluded in the main analysis due to health workers’ strike

^a^Chonyi was used in the correlates of protection models only

For the parasitaemia determination by microscopy, the number of asexual-stage parasites/200 leukocytes was counted, and parasitaemia was estimated based on actual or assumed (8,000 leukocytes/µL) leukocyte count measured for each blood smear.

### Statistical analysis

A comparison of logistic regression and Bayesian latent class models as estimators of malaria positivity was done. For both approaches, the relationship between the risk of fever and parasite density was carried out separately for each year and age group. A parasite density cut-off was estimated from the logistic approach and probabilities of children with different levels of parasitaemia were estimated from the Bayesian approach using R [35] and OpenBugs [36] respectively. The malaria positivity estimates from the two approaches were investigated by comparing their statistical performance in selecting parameters of malaria protection. Specific to logistic models, the selected parasite density cut-off was used to define cases and controls.

### Logistic regression

A logistic regression model was fit to the data, modelling the risk of fever as a continuous function of the parasite density. The model was of the form ${\text{logit}}\left( {\pi_{i} } \right) = \alpha + f\left( {x_{i} } \right)$ where $\pi_{i}$ is the probability that observation $i$ with parasite density $x_{i}$ is a (fever) case. Along with $f\left( {x_{i} } \right) = \beta x_{i}^{\tau }$, a smooth monotonic function of $x^{\tau }$ where $\tau$ is the power transformation of the parasite density. This power function $\tau$ was tested at different values between 0.10 and 0.90 with a precision of 0.01 and the value that maximized the log-likelihood best was chosen. The malaria attributable fraction (MAF),$\lambda$, was estimated using the slope coefficient of the logistic regression; $\lambda = \left( {{1 \mathord{\left/ {\vphantom {1 N}} \right. \kern-\nulldelimiterspace} N}} \right){{\sum\limits_{1}^{i} {\left( {R_{i} - 1} \right)} } \mathord{\left/ {\vphantom {{\sum\limits_{1}^{i} {\left( {R_{i} - 1} \right)} } {R_{i} }}} \right. \kern-\nulldelimiterspace} {R_{i} }}$ where $R_{i} = \exp \left[ {f\left( {x_{i} } \right)} \right]$ and the standard error was estimated using the bootstrap approach with 1000 bootstrap samples [1].

### Bayesian latent class

For the Bayesian latent class model, the parasite density was resolved to a mixture of two multinomial distributions. One component $g_{1} (.)$ corresponds to non-malaria fever episodes and the other component, $g_{2} (.)$ to children with clinical malaria episodes (fever and parasites). Parasite levels during the cross-sectional bleed were available and were used as the training sample, i.e., a sample that comes from the component of the mixture corresponding to children without fever but who may have parasites. The data was then divided into $K$ ordered categories over the range of the parasite density ${\mathbf{x}}$. This was followed by counting the of test samples ${\mathbf{n}} = \left( {n_{0} ,n_{1} ,...,n_{k - 1} } \right)$ and control samples (non-fever cases) ${\mathbf{m}} = \left( {m_{0} ,m_{1} ,...,m_{k - 1} } \right)$. Then the MAF,$\lambda$, was then estimated from the two multinomial distributions,

1.1 $$\begin{gathered} \theta_{i} = P\left( {x \in {\text{ category i | }}P_{1} } \right), \hfill \\ \phi_{i} = P\left( {x \in {\text{ category i | }}P_{2} } \right), \hfill \\ \lambda = P\left( {x \in P_{2} } \right) \hfill \\ \end{gathered}$$

The parameters $P_{1}$ and $P_{2}$ are the distributions functions of the components $g_{1} (.)$ and $g_{2} (.)$ respectively. The category-specific attributable fractions were obtained using,

1.2 $$\lambda_{i} = P\left( {x \in P_{2} { | }x \, \in {\text{category i }}} \right) = \frac{{\lambda \phi_{i} }}{{\left( {1 - \lambda } \right)\theta_{i} + \lambda \phi_{i} }}.$$

To estimate the probability, $\lambda_{ind}$, of each individual case of fever being attributable to malaria local and piece-wise cubic polynomial models were used. The models were fitted using category-specific MAF, $\lambda_{c}$, together with the category-specific midpoint of parasite density. This was followed by predicting the individual $\lambda_{ind}$ using their parasite density measurements from the results of various model fitting functions.

Sensitivity and specificity of various cut-off values for parasite density were estimated by $${\raise0.7ex\hbox{${n_{c} \lambda_{c} }$} \!\mathord{\left/ {\vphantom {{n_{c} \lambda_{c} } {N\lambda }}}\right.\kern-\nulldelimiterspace} \!\lower0.7ex\hbox{${N\lambda }$}}$$ and $${\raise0.7ex\hbox{${1 - n_{c} \left( {1 - \lambda_{c} } \right)}$} \!\mathord{\left/ {\vphantom {{1 - n_{c} \left( {1 - \lambda_{c} } \right)} {N\left( {1 - \lambda } \right)}}}\right.\kern-\nulldelimiterspace} \!\lower0.7ex\hbox{${N\left( {1 - \lambda } \right)}$}}$$ respectively where $$n_{c} = \sum\nolimits_{i = c}^{K} {n_{i} }$$,$$\lambda_{c} = {\raise0.7ex\hbox{${\left( {\sum\nolimits_{i = c}^{K} {\lambda_{i} n_{i} } } \right)}$} \!\mathord{\left/ {\vphantom {{\left( {\sum\nolimits_{i = c}^{K} {\lambda_{i} n_{i} } } \right)} {n_{c} }}}\right.\kern-\nulldelimiterspace} \!\lower0.7ex\hbox{${n_{c} }$}}$$, $$n_{i}$$ the number of fever cases in the category $$i$$ and $$c$$ represents the parasite density category of which it is the selected cut-off in logistic regression or the lower bound for the category in latent class models. Specific to logistic estimation, cases were febrile children exceeding the selected cutoff and controls otherwise.

### Association with protection

Multi-level logistic and zero-inflated models were used to investigate the association between high versus low merozoite antibodies and clinical malaria. Various antibody concentrations were applied as cutoffs to define the high and low responders [26]. The results were used to compare the performance of probability and binary outcomes. Since there was a probability mass at zero due to non-febrile participants, the zero inflated modelling approach was utilized. Specifically, for the probability outcome, results from the Maximum Likelihood (MLE) and Bayesian inference estimations were compared [26, 37, 38].

## Results

### Study population

A total of 268 participants from Chonyi and 4722 participants from Junju, Kilifi County were recruited in 2000 and from 2005 to 2017 respectively. Approximately 300 or more participants were followed up each year with average recruitment age of 6.5 years (ranging between 1 month old to 16 years) as shown in Table 1. Each child had on average 2.94 test occurrences during follow-up giving rise to a total of 14,404 events during the entire study period.

### Temporal distribution

Table 1 shows the distribution of fevers (axillary temperature of ≥37.5 ℃) for the cross-sectional surveys. In Junju, approximately 1034 (2.19%) occasions of fever were reported during the cross-sectional surveys. A decreasing trend of fevers was observed over the study period except for 2005 and 2006 where the samples were collected specifically for a vaccine trial [25]. The prevalence of *Plasmodium falciparum* was also artificially high during this period since the participants were recruited during the malaria season. A decline in the prevalence of *P. falciparum* parasite was observed between 2006 to 2013 from 30.21% to 8.78%. This was followed by a slight increase in 2014 and 2015 then another decline in 2016 to 4.32% in 2017.

#### Relationship of fever to parasitaemia over time

**Fig. 2:**
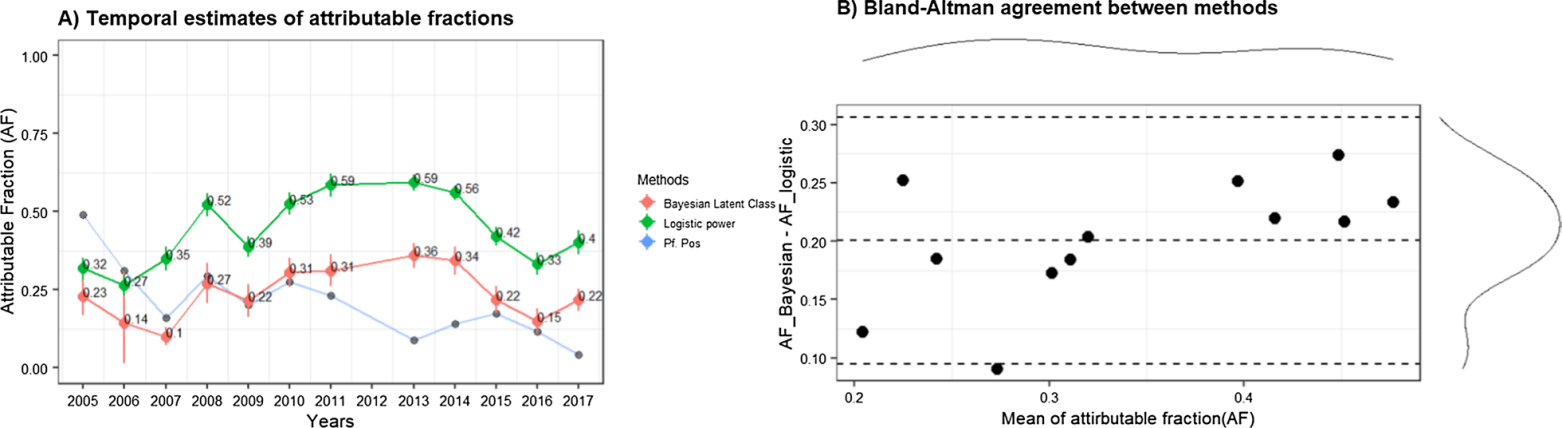
**A** Temporal estimates of attributable fraction (AF) from 2005 to 2017 using Bayesian latent class models and logistic power models. Pf. Pos is the prevalence of parasite positivity during the cross-sectional bleed. **B** Bland–Altman plot of agreement

The probability that a fever case was malaria attributable at a given parasite density $\lambda$ changed gradually over the study period as shown in Fig. 2. The MAF was estimated using the Bayesian latent class model and logistic regression using Junju cohort data only. The Bayesian latent class gave a lower MAF estimate, Bland–Altman bias = 0.20 (0.16–0.24), compared to the logistic model. After estimating the sensitivities and specifities of different parasite densities, the optimal parasite cut-off was selected using the logistic regression for the different years (Additional file 1: Table S1). However, the number of malaria positive individuals did not vary significantly with the new thresholds compared to the previously defined 2500p/µl threshold (Additional file 1: Fig. S1), despite the changing patterns. Notably, the Bayesian latent class approach and the logistic power models approximated a similar pattern of MAF but the estimates were lower in the former model. Comparable patterns were also observed in the probabilities,$\lambda_{i}$, predicted from the individual parasite densities (Additional file 1: Fig. S1).

#### Non-febrile individuals

An interval estimate for the prevalence of malaria fever was estimated using the Bayesian latent class model. The individual probabilities from the Bayesian fit for non-febrile participants with parasitaemia were adjusted using the interval estimate for the prevalence of malaria fever. This is shown in Additional file 1: Fig. S2 and Additional file 1: Fig. S3 where non-febrile cases had lower clinical malaria likelihood compared to the febrile cases for the parasite-positive individuals. Detailed implementation of the methodology is included in the repository as OpenBUGS and R codes.

#### Impact of age on MAF

**Fig. 3:**
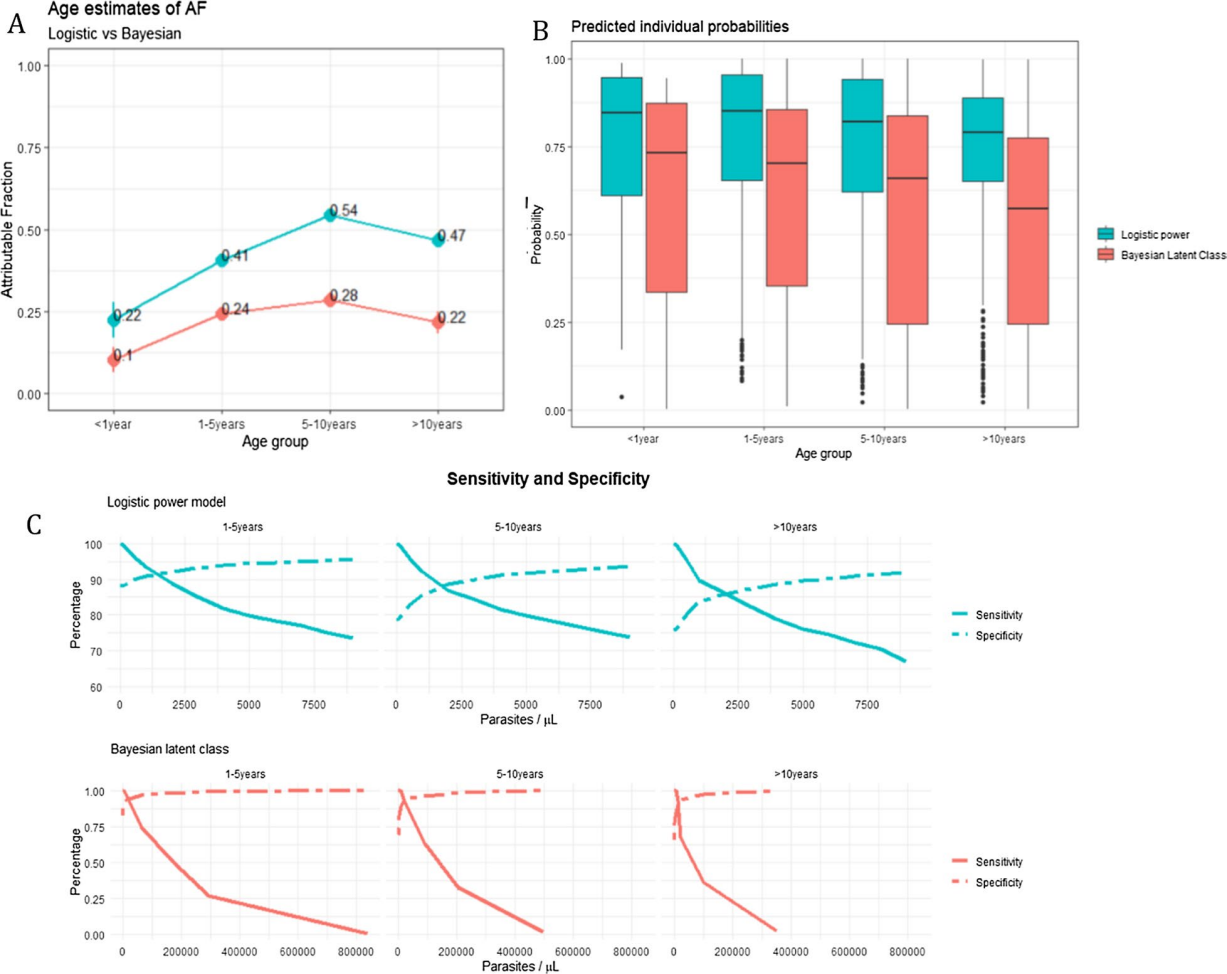
Malaria attributable fractions and probabilities over age group for all the study participants

The MAF estimates were higher for older age groups than the children < 1 year as shown in Fig. 3A. Additionally, the predicted individual probabilities declined with age as shown in Fig. 3B (Logistic power F = 12.63; p < 0.001 and Bayesian F = 18.95,p < 0.05) and likewise the logistic power model had higher estimates and a smaller range than the Bayesian latent class predictions Additional file 1: Table S2 and Additional file 1: Fig. S4. This shows as expected that the age groups of 1–5 years and 5–10 years had a higher probability of having malaria compared to the other age groups. The younger age groups had a higher specificity and sensitivity intersection (Fig. 3C) indicating a lower parasite density threshold for clinical episodes compared to the older age groups.

Predicted probabilities from Bayesian latent class model were compared with the binary outcome defined using logistic parasite density thresholds in identifying correlates of disease protection. To compare the perfomance, data on antibody responses to selected *P. falciparum* merozoite antigens for a study done in Kilifi was used. Specifically, the data had antibody measurements for the survey conducted in Junju in the year 2008 and a subset of the Chonyi cohort in the year 2000 [26].

A cut-off of 2500 parasites/µl Additional file 1: Table S1 plus fever was used to define the binary outcome (malaria positive, parasites $\ge$ 2500 parasites/µL or negative otherwise). The less predicted probabilities from Bayesian latent class models were used as the response variable to fit the zero–one inflated beta regressions. Table 2 shows that using the probability as the outcome gave comparable point estimates with the binary outcome. The binomial multilevel models, however, had high standard errors and Bayesian Information Criterion (BIC) values.

**Table 2:** A comparison of a binary and probability outcome using high vs low antibody levels in Junju 2008 and Chonyi 2000 cohort

		Binary outcome		Probability outcome		Probability Bayesian
		Coef. (SE)	BIC	Coef. (SE)	BIC	Coef. (SE)
Junju Cohort	AMA1	−0.30 (0.32)	427.73	−0.22 (0.20)	416.026	−0.23 (0.19)
	MSP2	0.09 (0.34)	428.6	−0.04 (0.20)	417.3474	−0.04 (0.21)
	MSP3	−0.24 (0.35)	428.18	−0.16 (0.19)	415.947	−0.19 (0.21)
Chonyi Cohort	AMA1	0.18 (0.34)	426.8972	−0.24 (0.11)	390.0447	−0.26 (0.13)
	MSP2	−0.67 (0.54)	425.4389	−0.33 (0.14)	401.3226	−0.34 (0.15)
	MSP3	0.68 (0.35)	423.5147	−0.11 (0.12)	410.5933	−0.12 (0.14)

*Coef* Regression Coefficients, *SE* Standard Error, *BIC* Bayesian Information Criterion

## Discussion

In areas with high malaria transmission, differences in the prevalence of malaria fever can occur due to change in transmission intensities or differences in levels of immunity in various subsets of the population like age groups [2]. The present study shows a variation of transmission intensity over time, and how this contributes to variation in the MAF. Similarly, a previous study done in Kilifi showed that immunity to malaria is affected by age and transmission [2]. The study compared Chonyi, a high transmission area and Ngerenya, a low transmission area. The sites had a variable age-specific clinical disease pattern with Ngerenya having a higher MAF compared to Chonyi overall and specifically for the older age group of 5-19 years. Shifting MAF was also observed with changing transmission patterns in the current study. A shift in malaria transmission intensities and malaria epidemiology has been reported in different endemic areas [14, 27].

Therefore, it is important to review MAF and case definitions with changing transmission settings. Furthermore, transmission intensity correlates with the rate of acquisition of natural immunity [28]. A decrease in malaria transmission intensity led to reduced immunity which would result in a higher tendency to acquire malaria attributable fevers at lower parasite densities as was observed in this study.

A strong rationale for developing malaria vaccines comes from cohort studies, which show that individuals continuously exposed to malaria develop immunity that initially prevents death from severe disease, and subsequently recurrent illness [12, 29]. The main assumption in defining correlates of protection to inform vaccine development is that malaria case definition is non-biased. Many of the studies classify the participants into two groups (clinical malaria case and non-case) using a defined parasitaemia threshold plus fever [1, 2, 7–9]. The optimal parasite density threshold is selected from maximum combined sensitivity and specificity after fitting the case definition models [1]. Additionally, the models estimate the probabilities individual episodes of fever are malaria attributable at a given density of parasitaemia [1, 3, 17].

The logistic power model is the widely used technique for case definition [2, 7–9, 30] and rarely the Bayesian latent class model [3, 17]. However, in this study the logistic approach was observed to give higher but comparable pattern estimates with the Bayesian latent class model. Similarly, this was observed in a study done by Vounatsou et al. comparing the logistic power and Bayesian latent class model [3]. The logistic model approach, however, has been reported to have a limitation of estimating imprecise standard errors and negative probabilities sometimes [5]. Comparatively, in this present study, the logistic approach also gave high probability estimates with narrow variation in the low parasite densities compared to the latent class model. In vaccine studies, the latent class was reported to help in identifying possible biases in efficacy estimates since it utilizes the whole range of possible parasite density cut-offs [19]. An inverse relationship of clinical malaria and age has been shown [11, 31], similarly, this was observed with the estimated probabilities which decreased with age.

Continuous variables, like the probabilities used here have been shown to have more variation information and statistical power and are sometimes preferred over the categorization of data [22]. Several articles [20–23] have also pointed out problems associated with the categorization of data. This study, compares the performance of using probability and binary outcome model the association with clinical malaria [26]. Assuredly, the probability model had a good statistical fit; lower BIC estimates and standard errors and gave comparable coefficients with the binary model. Also, the point estimates were similar to what was reported by Murungi et. al in the 2008 study using the same cohort [26], in which they reported risk ratios estimated using a modified Poisson regression [32]. This study however, reports coefficients showing a lower probability of disease for individuals with high antibody measurements.

## Strengths and limitation

For this study bi-weekly active surveillance was conducted. Therefore, short-lived asymptomatic infections below the level of detection by microscopy and exposure that does not result in a blood stage infection may have been missed. Parasite density cut-offs plus fever are used mostly in malaria endemic studies to inform policy. This study examined whether the varying intensity of malaria transmission affected the estimation of optimal cutoffs using the Junju cohort. Varying thresholds estimates were observed; however, this did not have a substantial impact on the number of febrile malaria individuals in this study. This research demonstrates the statistical advantage of utilizing probability outcomes over parasite thresholds. It has been shown that continuous variables, like the probabilities used, have more variation information and statistical power. Sometimes this is preferred over the categorization of data, however, a training dataset is required to estimate the probabilities [22].

In the malaria attributable fraction estimation, this study assumed independence of the malaria episodes of individuals with repeated measurements over the years. This was a major limitation; however, this was handled by considering the first 6 months of follow-up for the study participants per year of recruitment to reduce inter-dependence.

## Conclusion

The present study compares the performance of the logistic and Bayesian models in estimating MAF. Utilization of probabilities estimated from the Bayesian estimator has a better statistical fit in modelling the association of correlates of disease compared to the dichotomization approach of cases and controls using parasite thresholds from the logistic estimator. Another objective was to investigate whether the varying intensity of malaria transmission had a significant impact on parasite density thresholds.

Results from Junju and Chonyi cohorts verify the validity of using the probability outcome to identify correlates of disease protection while still having a better statistical fit. The computational time to fit the zero-inflated models was higher compared to the binary-based regression models and a training class is required for the latent class models which can be a limitation for some cohort designs.

Approaches to estimating an individual’s marginal probabilities of clinical malaria over a given follow-up time would be of importance for creating parsimonious models. Further studies to compare the probabilities estimated from models utilizing the quantitative nature of the parasite densities without grouping the data in conjunction with changing transmission would be valuable.

## Supplementary Information

**Additional file 1: Table S1.** Parasites/µL cut off using Logistic regression. **Fig S1.** Distribution of predicted probabilities. **Fig S2.** Comparison of probabilities for Bayesian and Logistic, Junju 2008. **Table S2.** Anova test for Figure 2B. **Fig S3.** Comparison of probability of febrile and non-febrile. **Fig S4.** Predicted probabilities over age groups. **Fig S5.** Sensitivity and specificity of the different years. **Fig S6.** Posterior estimates of AF.

## Data Availability

The datasets used and/or analysed during the current study are available from the corresponding author upon reasonable request. The scripts used for the current study are available in the GitHub repository, https://github.com/Keniajin/case_definition.
