# Supplementary material for: Malaria attributable fractions with changing transmission intensity: Bayesian latent class vs logistic models
Source: Malar J. 2022 Nov 11;21:326. doi: 10.1186/s12936-022-04346-9 (PMC9652045; doi:10.1186/s12936-022-04346-9)
Supplement: Supplementary file 1 — Additional file 1: Table S1. Parasites/µL cut off using Logistic regression. Fig S1. Distribution of predicted probabilities. Fig S2. Comparison of probabilities for Bayesian and Logistic, Junju 2008. Table S2. Anova test for Figure 2B. Fig S3. Comparison of probability of febrile and non-febrile. Fig S4. Predicted probabilities over age groups. Fig S5. Sensitivity and specificity of the different years. Fig S6. Posterior estimates of AF. [file 12936_2022_4346_MOESM1_ESM.pptx]

## Slide 1
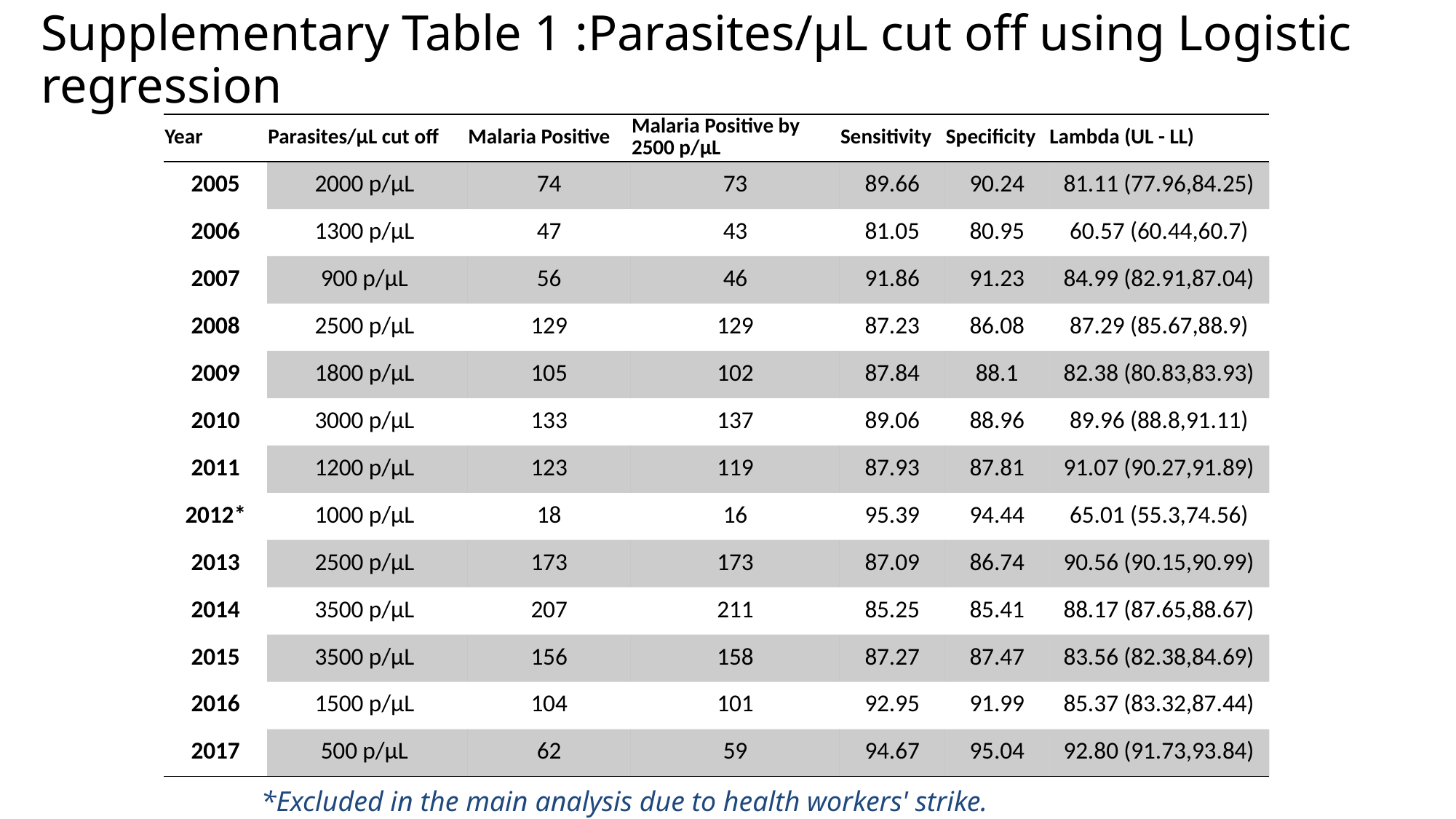

# Supplementary Table 1 :Parasites/µL cut off using Logistic regression
| Year | Parasites/µL cut off | Malaria Positive | Malaria Positive by 2500 p/µL | Sensitivity | Specificity | Lambda (UL - LL) |
| --- | --- | --- | --- | --- | --- | --- |
| 2005 | 2000 p/µL | 74 | 73 | 89.66 | 90.24 | 81.11 (77.96,84.25) |
| 2006 | 1300 p/µL | 47 | 43 | 81.05 | 80.95 | 60.57 (60.44,60.7) |
| 2007 | 900 p/µL | 56 | 46 | 91.86 | 91.23 | 84.99 (82.91,87.04) |
| 2008 | 2500 p/µL | 129 | 129 | 87.23 | 86.08 | 87.29 (85.67,88.9) |
| 2009 | 1800 p/µL | 105 | 102 | 87.84 | 88.1 | 82.38 (80.83,83.93) |
| 2010 | 3000 p/µL | 133 | 137 | 89.06 | 88.96 | 89.96 (88.8,91.11) |
| 2011 | 1200 p/µL | 123 | 119 | 87.93 | 87.81 | 91.07 (90.27,91.89) |
| 2012\* | 1000 p/µL | 18 | 16 | 95.39 | 94.44 | 65.01 (55.3,74.56) |
| 2013 | 2500 p/µL | 173 | 173 | 87.09 | 86.74 | 90.56 (90.15,90.99) |
| 2014 | 3500 p/µL | 207 | 211 | 85.25 | 85.41 | 88.17 (87.65,88.67) |
| 2015 | 3500 p/µL | 156 | 158 | 87.27 | 87.47 | 83.56 (82.38,84.69) |
| 2016 | 1500 p/µL | 104 | 101 | 92.95 | 91.99 | 85.37 (83.32,87.44) |
| 2017 | 500 p/µL | 62 | 59 | 94.67 | 95.04 | 92.80 (91.73,93.84) |
*Excluded in the main analysis due to health workers' strike.

## Slide 2
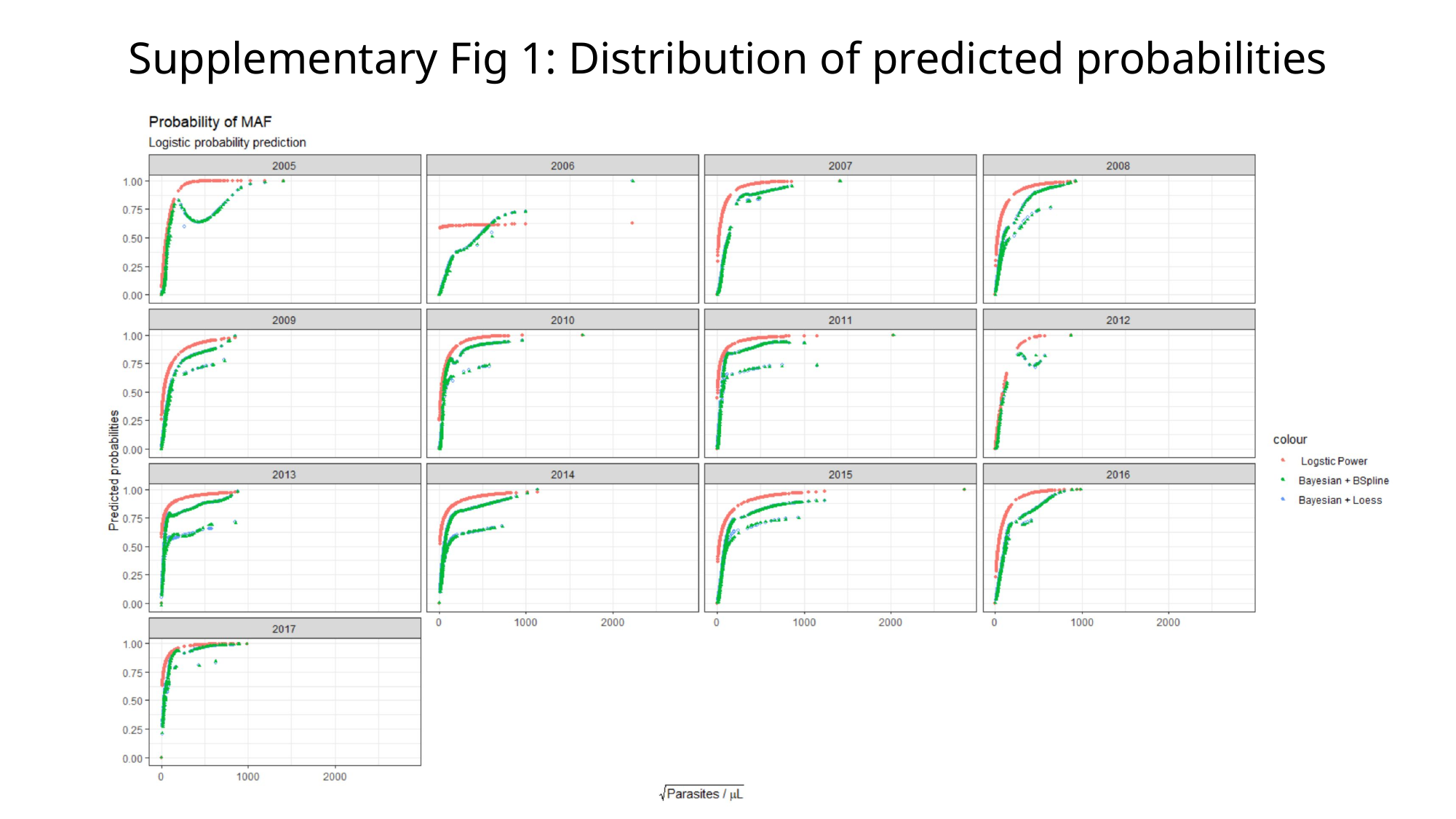

# Supplementary Fig 1: Distribution of predicted probabilities

## Slide 3
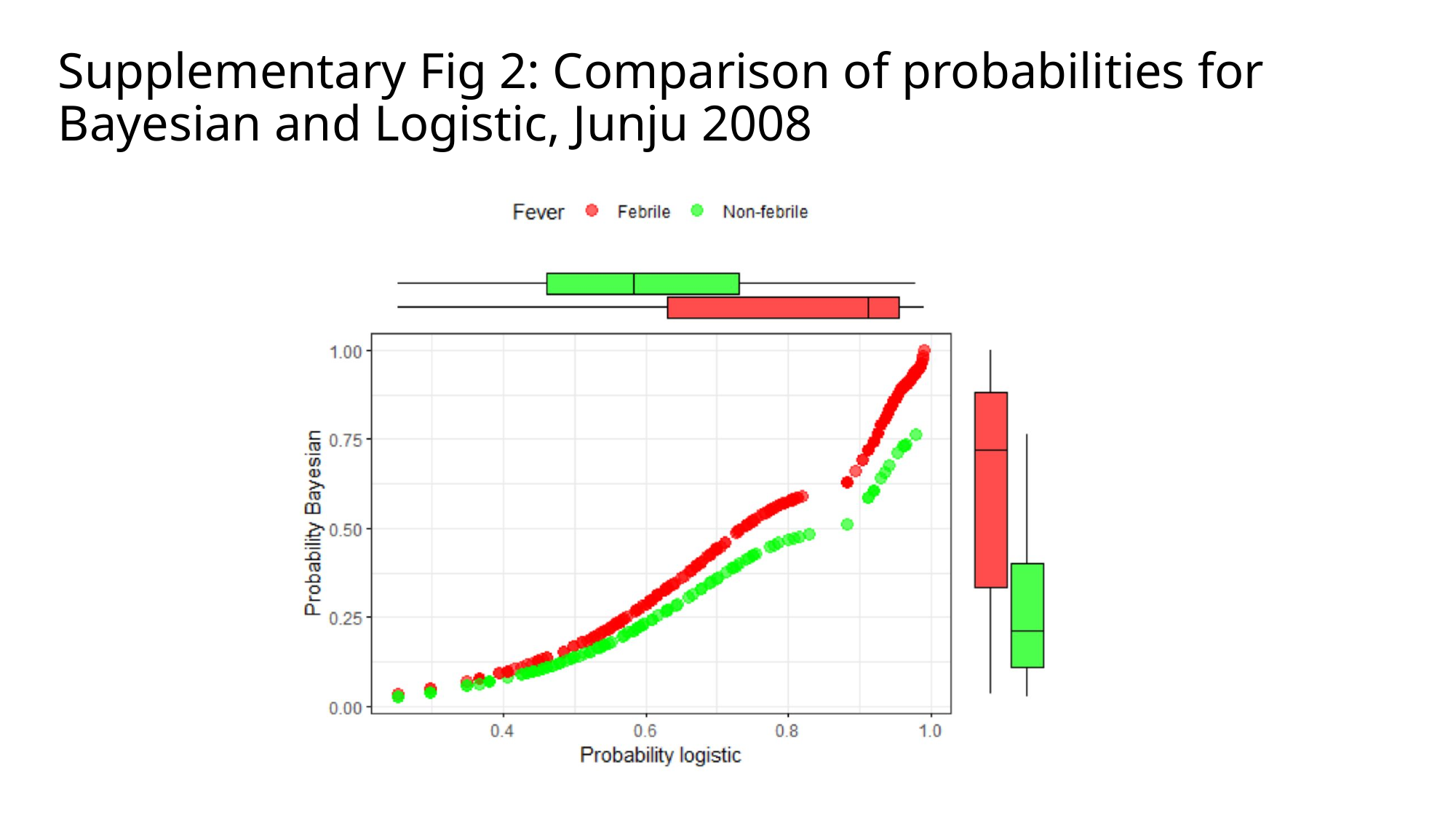

# Supplementary Fig 2: Comparison of probabilities for Bayesian and Logistic, Junju 2008

## Slide 4
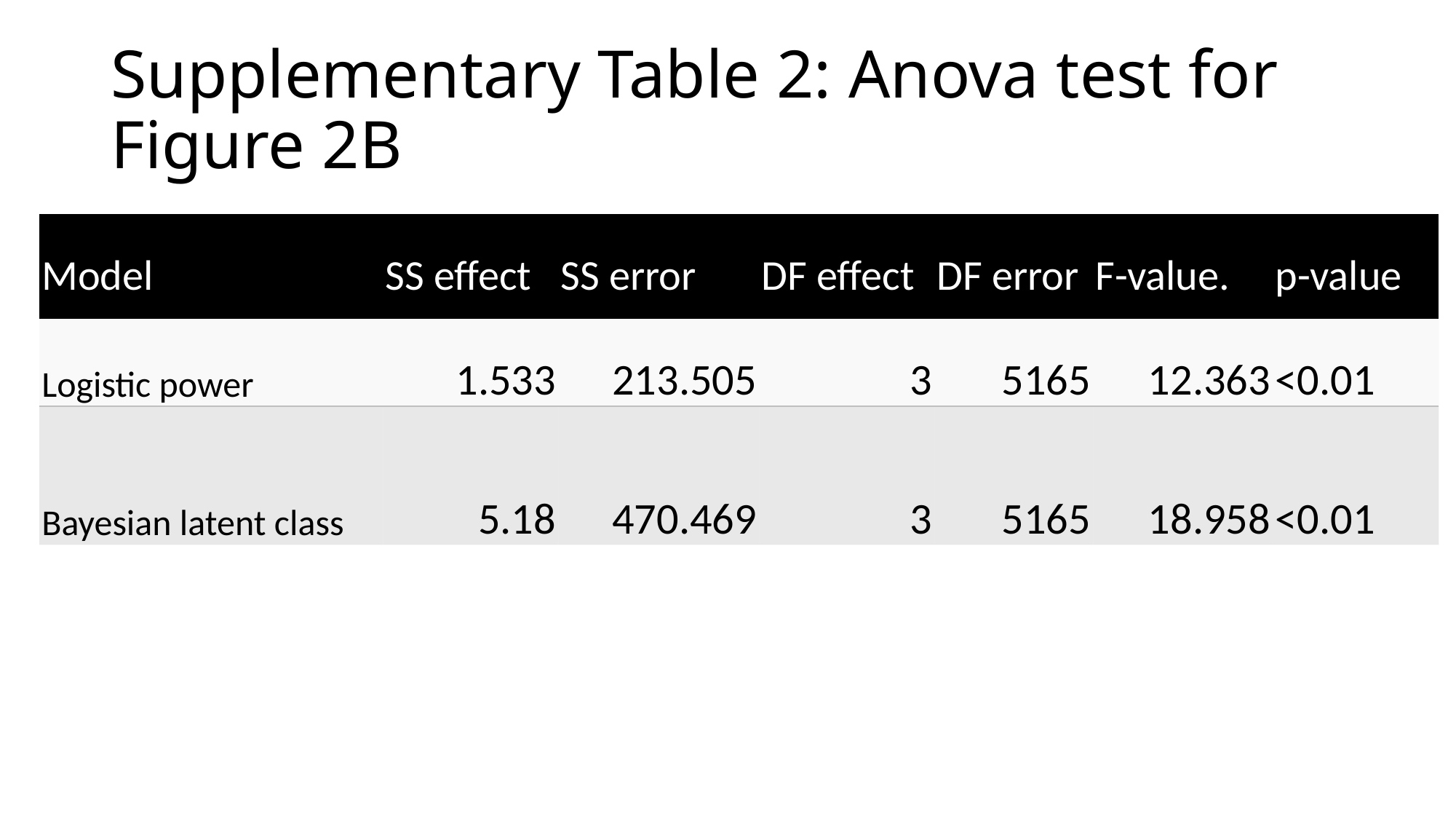

# Supplementary Table 2: Anova test for Figure 2B
| Model | SS effect | SS error | DF effect | DF error | F-value. | p-value |
| --- | --- | --- | --- | --- | --- | --- |
| Logistic power | 1.533 | 213.505 | 3 | 5165 | 12.363 | <0.01 |
| Bayesian latent class | 5.18 | 470.469 | 3 | 5165 | 18.958 | <0.01 |

## Slide 5
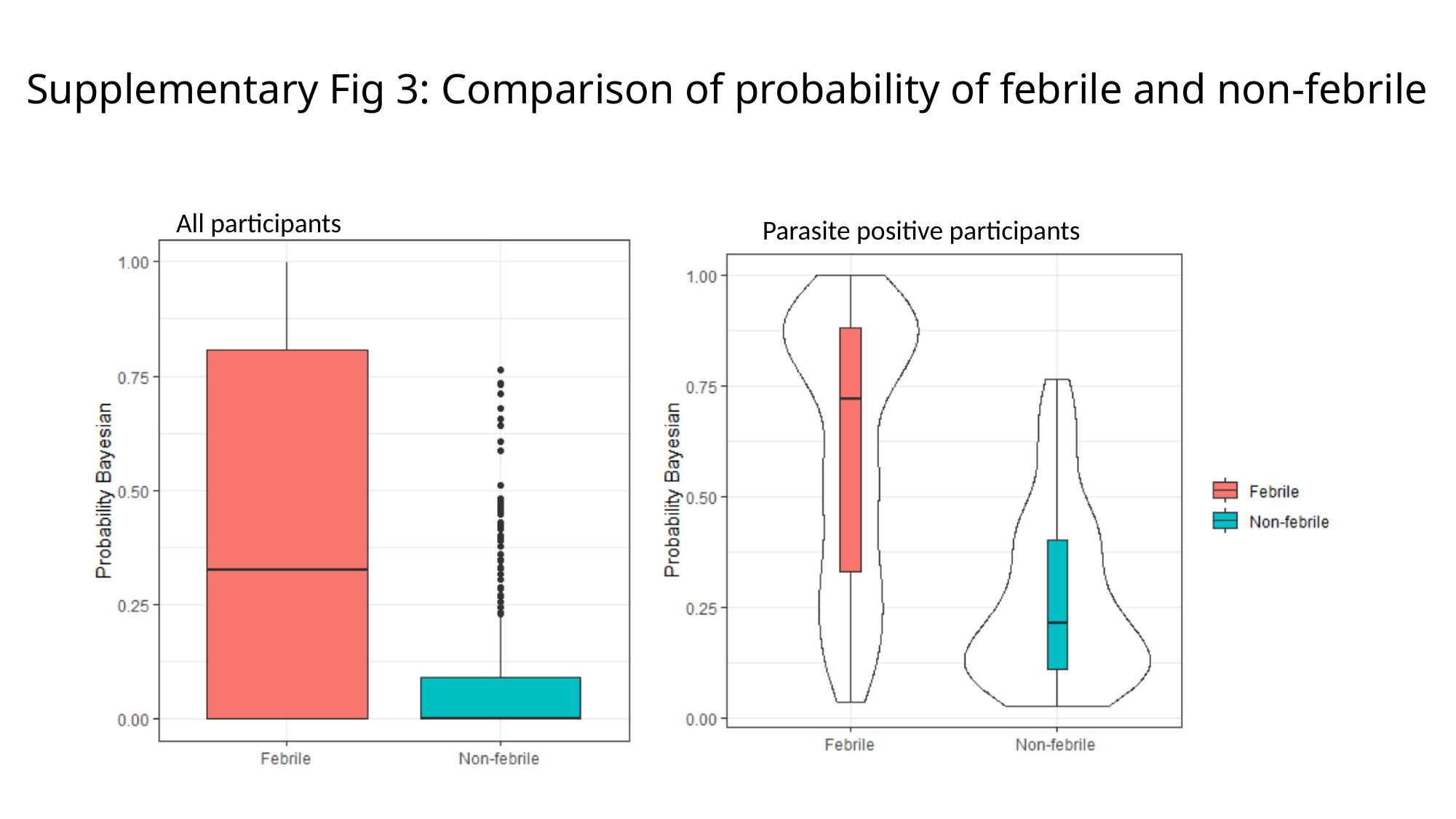

# Supplementary Fig 3: Comparison of probability of febrile and non-febrile
All participants
Parasite positive participants

## Slide 6
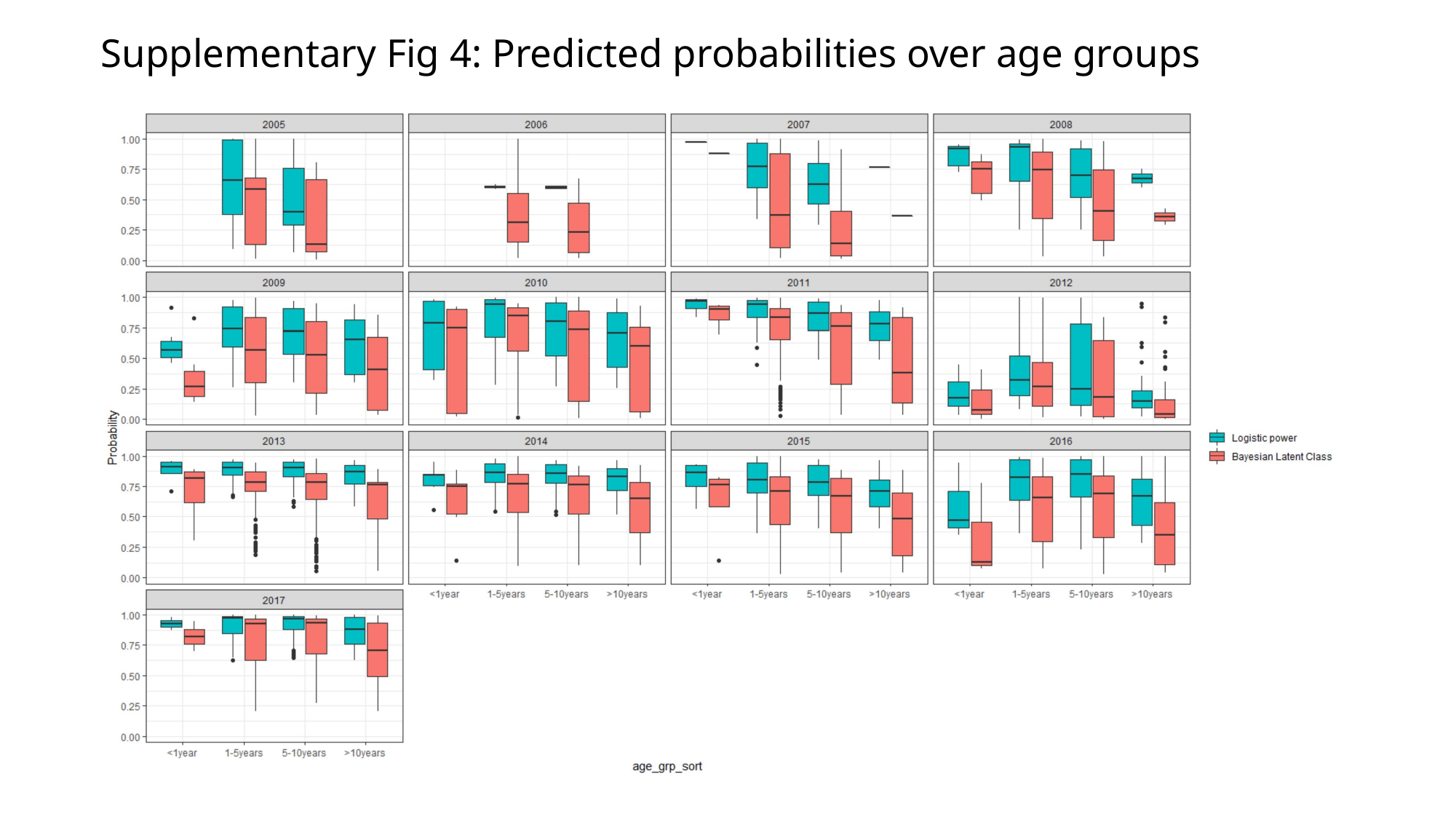

# Supplementary Fig 4: Predicted probabilities over age groups

## Slide 7
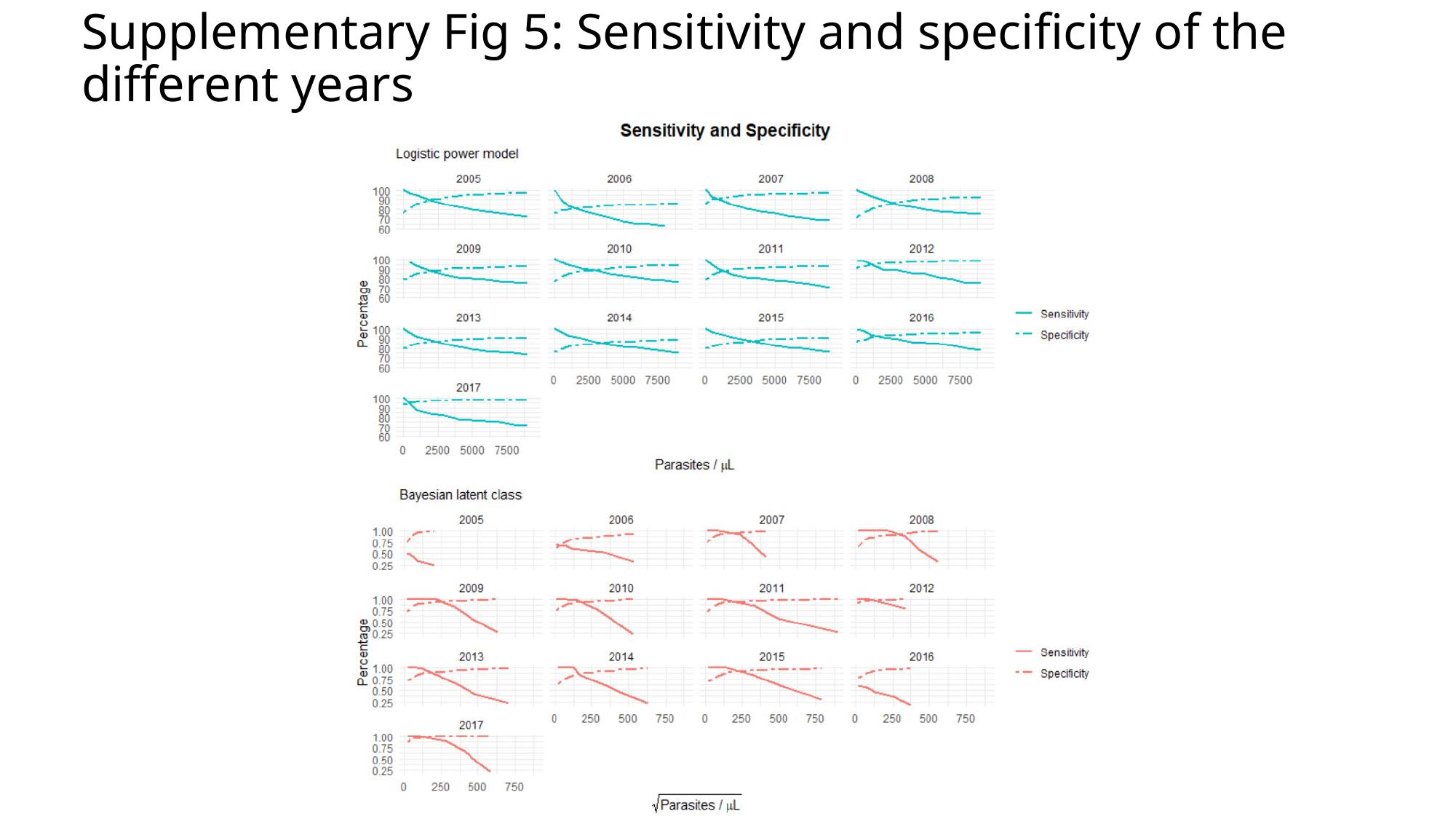

# Supplementary Fig 5: Sensitivity and specificity of the different years

## Slide 8
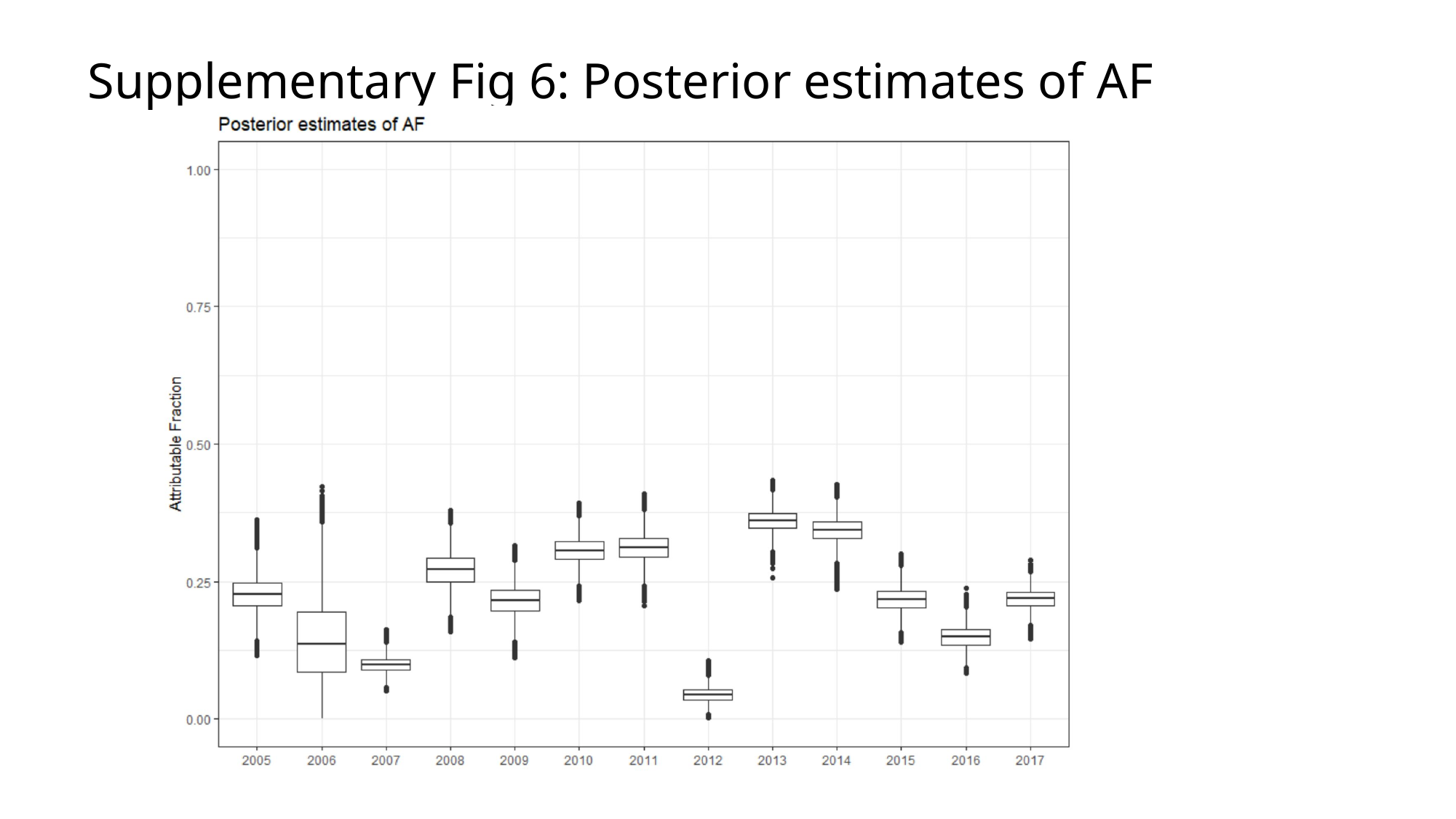

# Supplementary Fig 6: Posterior estimates of AF
